# Impact of prenatal exposure to cadmium on cognitive development at preschool age and the importance of selenium and iodine

**DOI:** 10.1007/s10654-016-0151-9

**Published:** 2016-05-04

**Authors:** Maria Kippler, Matteo Bottai, Vaggelis Georgiou, Katerina Koutra, Georgia Chalkiadaki, Mariza Kampouri, Andriani Kyriklaki, Marina Vafeiadi, Eleni Fthenou, Maria Vassilaki, Manolis Kogevinas, Marie Vahter, Leda Chatzi

**Affiliations:** 1 Institute of Environmental Medicine, Karolinska Institutet, Box 210, 171 77 Stockholm, Sweden; 2 Department of Social Medicine, Faculty of Medicine, University of Crete, 71003 Heraklion, Crete Greece; 3 Centre for Research in Environmental Epidemiology (CREAL), 08003 Barcelona, Spain; 4 Municipal Institute of Medical Research (IMIM-Hospital del Mar), 08003 Barcelona, Spain; 5 National School of Public Health, 11521 Athens, Greece; 6 Department of Genetics and Cell Biology, Faculty of Health, Medicine and Life Sciences, Maastricht University, Universiteitssingel 40, PO Box 616, 6229 ER Maastricht, The Netherlands

**Keywords:** Cognitive development, Prenatal exposure, Urine, Cadmium, Selenium, Iodine

## Abstract

The evidence regarding a potential link of low-to-moderate iodine deficiency, selenium status, and cadmium exposure during pregnancy with neurodevelopment is either contradicting or limited. We aimed to assess the prenatal impact of cadmium, selenium, and iodine on children’s neurodevelopment at 4 years of age. The study included 575 mother–child pairs from the prospective “Rhea” cohort on Crete, Greece. Exposure to cadmium, selenium and iodine was assessed by concentrations in the mother’s urine during pregnancy (median 13 weeks), measured by ICPMS. The McCarthy Scales of Children’s Abilities was used to assess children’s general cognitive score and seven different sub-scales. In multivariable-adjusted regression analysis, elevated urinary cadmium concentrations (≥0.8 µg/L) were inversely associated with children’s general cognitive score [mean change: −6.1 points (95 % CI −12; −0.33) per doubling of urinary cadmium; corresponding to ~0.4 SD]. Stratifying by smoking status (*p* for interaction 0.014), the association was restricted to smokers. Urinary selenium was positively associated with children’s general cognitive score [mean change: 2.2 points (95 % CI −0.38; 4.8) per doubling of urinary selenium; ~0.1 SD], although the association was not statistically significant. Urinary iodine (median 172 µg/L) was not associated with children’s general cognitive score. In conclusion, elevated cadmium exposure in pregnancy of smoking women was inversely associated with the children’s cognitive function at pre-school age. The results indicate that cadmium may adversely affect neurodevelopment at doses commonly found in smokers, or that there is an interaction with other toxicants in tobacco smoke. Additionally, possible residual confounding cannot be ruled out.

**Electronic supplementary material:**

The online version of this article (doi:10.1007/s10654-016-0151-9) contains supplementary material, which is available to authorized users.

## Introduction

Millions of children worldwide never reach their full neurodevelopmental potential. Simultaneously, the numbers of environmental chemicals that have been associated with neurodevelopmental toxicity are steadily increasing [1, 2]. Toxic trace elements, such as lead, methylmercury, arsenic and manganese have repeatedly been shown to impair neurodevelopment [2, 3]. However, evidence concerning the potential effect of cadmium, a highly toxic metal present in food and tobacco smoke, remains conflicting [3].

A systematic review and meta-analysis [3], covering studies between January 2000 and March 2012, identified four studies exploring an association between cadmium exposure and neurodevelopment, but only one study had measured the prenatal exposure. This Chinese study, found an inverse association between the children’s prenatal cadmium exposure, assessed by concentrations in cord blood, and full scale IQ at 4.5 years [4]. Thereafter, a study of 1305 Bangladeshi mother–child pairs found that maternal urinary cadmium concentrations during pregnancy were inversely associated with their children’s full scale, performance, and verbal IQ at 5 years of age [5]. In a South Korean mother–child cohort, maternal blood cadmium concentrations during pregnancy were not inversely associated with neurodevelopment at 6 months (n = 884) [6], but at a follow-up at 5 years of age (n = 119), maternal blood concentrations were inversely associated the children’s performance IQ [7]. In a Spanish study (n = 485), no association was observed between prenatal cadmium exposure and neurodevelopment at 4 years of age [8]. The conflicting findings may be a result of low power in several studies, the use of different developmental tests, and differences in diet and nutritional status.

Iodine is essential for the production of thyroid hormones [thyroxine (T4) and triiodothyronine (T3)] [9], and thus, severe iodine deficiency during pregnancy has been linked with impaired neurodevelopment in the offspring [10]. More recent studies have assessed if mild-to-moderate iodine deficiency may also affect neurodevelopment, but so far the results are inconsistent [11, 12]. Selenium has also been shown to interact with thyroid metabolism [13], but studies on selenium and early-life neurodevelopment are very limited. In a mother-child cohort in Bangladesh, we recently showed that women’s selenium status during pregnancy was positively associated with their children’s cognitive development at 1.5 years of age [14]. The aim of the present study was to evaluate the impact of prenatal exposure to cadmium, selenium, and iodine on Greek children’s neurodevelopment at 4 years. We evaluated the elements separately as well as when taking the other elements into account.

## Materials and methods

### Study population

The present study was conducted within the “Rhea study”, a prospective longitudinal mother–child cohort in Heraklion, Greece [15]. The mothers were recruited to the cohort in early pregnancy, in connection with the first ultrasound examination (before 15 weeks of gestation), and were contacted again for follow-up of the children at 9, 18 months, and 4 years of age. The inclusion criteria were confirmed pregnancy, residency within the study area, aged 16 years or older and good understanding of the Greek language. In total, 879 singleton children participated in the 4 years follow up of the study, during which cognitive and neuropsychological development was assessed in 785 children. From those, complete data for different toxic and essential elements (e.g. cadmium, selenium and iodine) during pregnancy and a follow-up interview was available for 628 mother–child pairs. We had to exclude 53 children due to incomplete information regarding their mothers age (n = 14), education (n = 7), civil status (n = 2), parity (n = 18), and tobacco smoking habits (n = 12). Thus, a cohort of 575 mother–child pairs (91 % of the children with prenatal exposure data and developmental assessment) was included in the present evaluation. We observed no difference in any of the exposures (*p* for all 0.060–0.62) or outcome scores (*p* for all 0.090–0.89) between the children that we included in the present analyses (n = 575) and those that were excluded due to incomplete covariate information (n = 53).

The present study was conducted according to the principles of the Helsinki Declaration, and it was approved by the ethical committee of the University Hospital in Heraklion, Greece, as well as the ethical committee at Karolinska Institutet, Sweden. Informed consent was obtained from all participants included in the present study.

### Exposure assessment

Spot urine samples collected from the women during pregnancy (median 13 weeks of gestation, interquartile range 4 weeks) were used to assess the children’s prenatal exposure to cadmium, selenium, and iodine. Urinary cadmium is a well-documented biomarker of chronic exposure [16]. Since the main route of excretion of selenium is via urine, urinary selenium has been suggested as a good biomarker of selenium status [17]. Similarly, iodine is also mainly excreted via urine and it is considered as a valid biomarker for assessment of iodine intake on population-basis [18].

Cadmium, selenium and iodine in urine were measured with inductively coupled plasma mass spectrometry (ICPMS; Agilent 7700×, Agilent Technologies, Tokyo, Japan), equipped with an octopole reaction system, at Karolinska Institutet, Sweden [19, 20]. When measuring cadmium and selenium the urine samples were diluted 1:10 in 1 % nitric acid (prepared from 65 % suprapur, Merck, Darmstadt, Germany). To minimize potential interferences, cadmium (isotope 111) was measured in helium mode, while selenium (isotope 78) was measured in hydrogen mode. For the measurements of iodine the urine samples were diluted 1:10 in 0.1 % ammonium hydroxide solution (NH_4_OH; 25 % suprapur, Merck, Darmstadt, Germany), and iodine (isotope 127) was measured in standard mode (no gas). The limit of detection (LOD) for urinary cadmium, selenium and iodine was <0.010, <0.014, and <0.10 µg/L, respectively. No samples contained concentrations below the calculated LODs. Quality control was performed by including either one or two commercial control materials (Seronorm™ Trace Elements Urine Blank, REF 201305, LOT OK4636 and Seronorm™ Trace Elements Urine, REF 201205, LOT NO2525) in each analytical run. In general, the obtained mean urinary concentrations of cadmium, selenium and iodine showed a good agreement with the recommended value of each element.

The urinary element concentrations were adjusted for the specific gravity (SG) in order to compensate for the variation in urine dilution (urinary concentration * [mean SG (1.020)−1/individual SG-1]) [21]. The specific gravity was measured with a digital refractometer (EUROMEX RD712 Clinical Refractometer, Holland).

### Neurodevelopmental assessment

Children’s neuropsychological development was assessed by two trained psychologists, with the McCarthy Scales of Children’s Abilities (MSCA), developed for children aged 2½–8½ years. In brief, the MSCA test aims to assess the children’s general cognitive function through five different sub-scales: verbal, quantitative, memory, perceptive-performance, and motor [22]. Executive function and cognitive functions of posterior cortex are two additional scales derived from reorganization of the MSCA subtests in accordance with their association with specific neurocognitive function areas [23, 24]. Raw scores of the neurodevelopmental assessment scales were standardized for child’s age at test administration using a method for the estimation of age-specific reference intervals based on fractional polynomials [25]. To harmonize the scales, the residuals were standardized to a mean of 100 points with a standard deviation (SD) of 15 (parameters conventionally used in psychometrics for IQ assessment). Higher scores represented better general cognitive function.

### Potential covariates

Personal interviews, together with self-administrated questionnaires and medical records, were used to obtain information regarding socio-demographic, environmental and psychosocial factors during pregnancy and childhood. The mothers’ characteristics included: education at recruitment (≤6 years of school, >6 to ≤12 years of school, and >12 years of school), origin (Greek or non-Greek), parity (nulliparous or multiparous) and body mass index (BMI; kg/m^2^) before pregnancy, employment (yes/no), tobacco smoking habits during pregnancy (never, quit during pregnancy, smoked during pregnancy), age (<25 years of age, ≥25 to <35 years of age, and ≥35 years of age) and civil status (married/engaged or single) at birth of the child. The children’s characteristics included: sex (male/female), gestational age at birth (weeks), weight (g) at birth, duration of breast feeding (no, 1–6 moths or >6 months), and age (years), height (cm), and weight (kg) at developmental assessment. The children’s weight and height measured at the developmental assessment were converted to weight-for-age (WAZ), height-for-age (HAZ), and body mass index-for-age (ΒΜΙ) z-scores (SD scores), using to the WHO Multicentre Growth Reference Study child growth standards [26].

### Statistical analyses

**Fig. 1:**
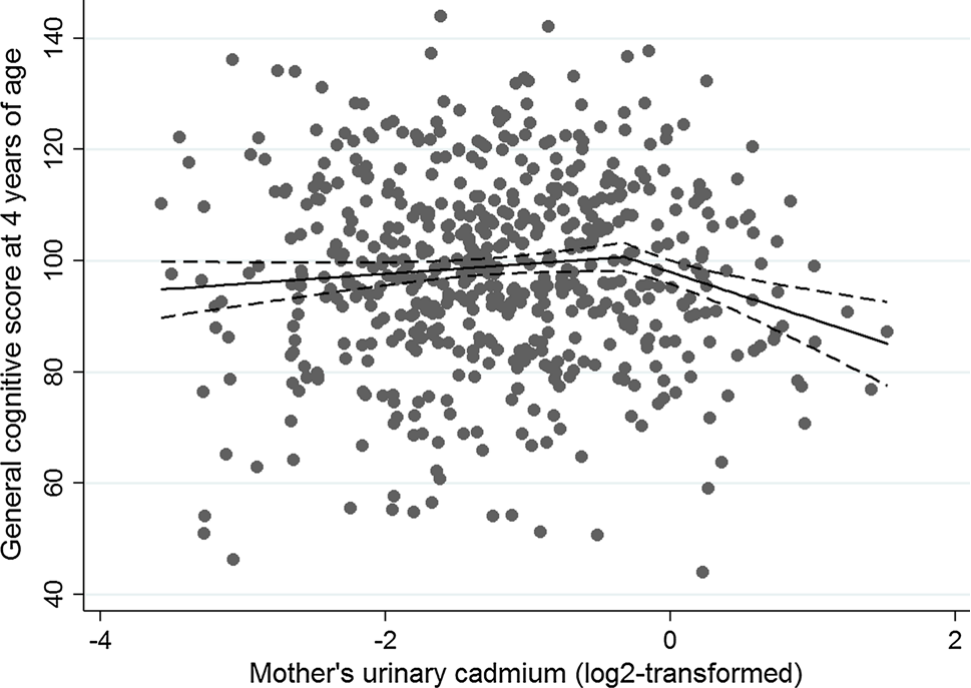
Association between mother’s urinary cadmium concentrations during pregnancy (log_2_-transformed) and children’s general cognitive scores at 4 years of age. The *solid line* represent the mean change in the general cognitive score for every doubling in the mother’s urinary cadmium concentration and the *dashed lines* the corresponding 95 % confidence intervals

We started with exploring whether the children’s prenatal exposure to cadmium (mother’s urinary cadmium concentration) or prenatal nutritional status of selenium and iodine (mother’s urinary selenium and iodine) was associated with their general cognitive score at 4 years of age by examining scatterplots with a moving average Lowess curve. The association between the mother’s urinary cadmium concentration and the children’s general cognitive score was nonlinear (Fig. 1); slightly positive up to a urinary concentration of 0.8 µg Cd/L, and steeply decreasing after that value. The associations of urinary selenium and urinary iodine with the children’s general cognitive score appeared linear. For explanatory purposes we also assessed associations of the biomarkers with the seven different sub-scales of the children’s cognitive score (i.e. verbal score, quantitative score, memory score, perceptive-performance score, motor score, and executive function and cognitive function scores). Crude associations between biomarkers, outcomes, and potential covariates were assessed using Spearman’s correlation coefficient. Differences between independent groups were determined by Mann–Whitney’s U test, analysis of variance, or the Chi square test depending on the type of data analyzed.

Further statistical analyses were performed using multivariable-adjusted linear regression analyses. To reduce the influence of some outliers in the upper concentration range, all the biomarkers were log_2_-transfomed. We chose log_2_-transformation to simplify the interpretation of the beta-coefficients in the linear regression analyses (mean change in outcome per every doubling of the biomarker). For urinary cadmium, we applied a spline knot at 0.8 µg Cd/L (on a log_2_-scale −0.32) to account for the non-linear association, obtaining two different beta coefficients in each model (β1 and β2). The models were adjusted for child sex, age at testing, and for the examiner who conducted the developmental testing. We also included covariates that were associated with any of the biomarkers and outcomes (*p* < 0.10; the mother’s age, parity, civil status, education, and tobacco smoking status). In Model 1, we assessed each biomarker (urinary cadmium, selenium, and iodine) separately, while in Model 2 all the biomarkers were included jointly. Model 3 was additionally adjusted for urinary lead, as lead is a well-known neurotoxicant [27].

In sensitivity analyses, we additionally adjusted Model 3 for potential mediating factors in the casual pathways between our prenatal exposures and the children’s general cognitive score such as birth weight, gestational age at birth (<37 and ≥37 weeks of gestation), duration of breastfeeding (no, 1–6, and >6 months), or children’s WAZ at 4 years of age. We also restricted the analyses to children born at term (≥37 weeks of gestation).

As previous studies have indicated sex-differences in the associations of cadmium and selenium with children’s development [5, 6, 14], we assessed modification by sex by including a multiplicative interaction term in Model 3 [biomarker * sex (boy = 0 and girls = 1)]. The same approach was used in relation to maternal smoking (never = 0 and ever = 1), as smoking is a well-known source of cadmium [16] and has previously been shown to modify selenium status [28] as well as child development [23]. In case the interaction term was significant (*p* values <0.10), we also performed stratified analyses.

The statistical analyses were performed with STATA (version 11, Statacorp TX, USA). When assessing different associations between biomarkers and outcomes a *p* value <0.05 were considered statistically significant, and for interactions *p* values <0.10 were considered statistically significant.

## Results

The mother’s showed a wide range of urinary cadmium concentrations during pregnancy, 0.084–2.9 µg/L, with a median of 0.43 µg/L (mean 0.54 µg/L). About 18 % of the mother’s (n = 106) had a urinary concentration equal to or above 0.8 µg Cd/L, and 10 % (n = 62) had a urinary cadmium concentration which exceeded 1.0 µg Cd/L. The mothers who either reported that they quit tobacco smoking during pregnancy or continued to smoke during their whole pregnancy period had slightly, although significantly, higher urinary cadmium concentrations (median 0.48 µg/L; 1–99th percentile 0.10–2.4 µg/L; n = 197) than those who reported that they had never smoked (median 0.40 µg/L; 1–99th percentile 0.096–1.7 µg/L; n = 378; *p* < 0.01). The median concentration of urinary selenium and iodine was 22 µg/L (range 7.7–86 µg/L) and 172 µg/L (range 30–797 µg/L), respectively. The mean age of the children at developmental testing was 4.2 years (SD ± 0.23) and 50 % were boys (Table 1). About 15 % of the children were born preterm (<37 week of gestation). As shown in Fig. 1, the mother’s urinary cadmium concentration was positively associated with the children’s general cognitive score up to a concentration of 0.8 µg Cd/L after which the association became negative. Urinary selenium was weakly positively associated with the children’s general cognition (r_s_ = 0.074; *p* = 0.076), while no association was observed between urinary iodine and general cognition (r_s_ = 0.015; *p* = 0.72).

**Table 1:** Characteristics of the mothers and their children at birth and 4 years of age

Variables	N	Mean ± SD or percent	Median	5–95th Percentile
*Maternal characteristics*				
Age (years)	575	30 ± 5.1	30	21–38
BMI early pregnancy (kg/m^2^)	575	25 ± 4.7	24	19–34
Education^a^	97/297/181	17/52/31		
Origin (Greek/non-Greek)	538/34	94/6		
Civil status (couple/single)	508/67	88/12		
Parity (nulliparous/multiparous)	239/336	42/58		
Smoking (never/ever)	378/197	66/34		
Urinary Cd (µg/L)^b^	575	0.54 ± 0.39	0.43	0.16–1.2
Urinary Se (µg/L)^b^	575	23 ± 8.6	22	12–39
Urinary iodine (mg/L)^b^	575	0.19 ± 0.092	0.17	0.087–0.36
Urinary Pb (µg/L)^b^	575	1.3 ± 1.1	1.1	0.32–2.5
*Child characteristics*				
Birth weight (g)	574	3180 ± 514	3200	2370–3980
Gestational age (weeks)	568	38 ± 1.6	38	35–40
Breast feeding (no/1–6/>6 months)	77/312/160	14/57/29		
Age at assessment (years)	575	4.2 ± 0.23	4.2	4.0–4.8
Weight at assessment (kg)	574	18 ± 3.0	18	14–24
Height at assessment (cm)	573	105 ± 4.3	105	98–113
General cognitive scores	575	98 ± 17	98	69–124
Verbal scores	575	98 ± 17	99	68–126
Quantitative scores	575	98 ± 17	98	68–123
Memory scores	575	98 ± 17	99	67–124
Perceptive-performance scores	575	99 ± 16	100	71–125
Motor scores	575	99 ± 17	100	72–124
Executive functions, frontal cortex	575	98 ± 17	100	69–125
Cognitive scores, posterior cortex	575	98 ± 17	99	70–125

^a^Years of schooling categorized as <6 years, ≥6 to ≤12 years, and >12 years

^b^Adjusted for specific gravity 1.020; median gestational week 13

In the multivariable-adjusted analyses (Table 2; Model 1), maternal urinary cadmium concentrations ≥0.8 µg/L (log_2_ transformed) during pregnancy were inversely associated with their children’s general cognitive score at 4 years of age (β2: −6.2; 95 % CI: −12, −0.54; *p* = 0.032). On the contrary, urinary cadmium concentrations <0.8 µg/L were not associated with the children’s general cognitive score (β: 1.1; 95 % CI: −0.83; 3.0; *p* = 0.27). After additional adjustment for urinary selenium and urinary iodine (Table 2; Model 2), the inverse association of maternal urinary cadmium ≥0.8 µg/L with the general cognitive score remained essentially the same (β2: −6.0; 95 % CI: −12, −0.27; *p* = 0.040). Similarly, additional adjustment for urinary lead did not change the association between cadmium and general cognition (Table 2; Model 3), and urinary lead was not associated with the children’s general cognitive score (β: −0.43; 95 % CI −1.5, 0.68; *p* = 0.45). In the fully adjusted model, at concentrations above 0.8 µg/L, a doubling of the mothers’ urinary cadmium was associated with a mean decrease in the children’s general cognitive function by 6.1 points (95 % CI: −12; −0.33; corresponding to about 0.4 SD). Urinary selenium was positively associated with the children’s general cognitive score (Table 2; Model 3), although the association was not significant (*p* = 0.095). A doubling in the mother’s urinary selenium increased the children’s general cognitive function by 2.2 points (95 % CI: −0.38; 4.8; corresponding to about 0.1 SD). Urinary iodine was not associated with the general cognitive scores in any of the models. In relation to different sub-scales of general cognition, urinary cadmium concentrations ≥0.8 µg/L were inversely associated the verbal score (*p* = 0.018), quantitative score (*p* = 0.040), and executive function score (*p* = 0.014; Table 2), while the positive association with urinary selenium was most pronounced for the verbal score (*p* = 0.085) and cognitive function score (*p* = 0.055).

**Table 2:** Regression analysis with maternal urinary concentrations of cadmium (spline knot at 0.8 µg Cd/L), selenium, and iodine (log_2_-tranfomed) in relation to their children’s general cognitive score, and by different sub-scales of the general cognitive score, at 4 years of age

Outcomes	Urinary Cd (log_2_; spline knot at 0.8 µg/L)				Urinary Se (log_2_)		Urinary iodine (log_2_)	
	β1 (95 % CI)^a^	*p*	β2 (95 % CI)^a^	*p*	β (95 % CI)	*p*	β (95 % CI)	*p*
*General cognitive scores*								
Model 1^b^	1.1 (−0.83; 3.0)	0.27	−6.2 (−12; −0.54)	0.032	1.9 (−0.57; 4.3)	0.13	0.12 (−1.8; 2.0)	0.91
Model 2^c^	0.60 (−1.3; 2.5)	0.55	−6.0 (−12; −0.27)	0.040	2.1 (−0.45; 4.7)	0.11	−0.18 (−2.1; 1.8)	0.86
Model 3^d^	0.74 (−1.2; 2.7)	0.46	−6.1 (−12; −0.33)	0.038	2.2 (−0.38; 4.8)	0.095	−0.13 (−2.1; 1.8)	0.90
*P* interaction^e^		0.55		0.40		0.13		
*P* interaction^f^		0.034		0.014		0.99		
*Verbal scores*								
Model 1^b^	1.7 (−0.25; 3.7)	0.087	−7.6 (−14; −1.4)	0.016	2.2 (−0.31; 4.7)	0.085	−0.053 (−2.0; 1.9)	0.96
Model 2^c^	1.2 (−0.74; 3.1)	0.22	−7.4 (−14; −1.2)	0.020	2.2 (−0.30; 4.8)	0.084	−0.35 (−2.3; 1.6)	0.73
Model 3^d^	1.5 (−0.54; 3.4)	0.15	−7.5 (−14; −1.3)	0.018	2.4 (−0.14; 5.0)	0.063	−0.26 (−2.3; 1.7)	0.79
*P* interaction^e^		0.62		0.28		0.17		
*P* interaction^f^		0.11		0.032		0.67		
*Quantitative scores*								
Model 1^b^	0.82 (−1.17; 2.8)	0.42	−6.0 (−12; −0.37)	0.037	−0.072 (−2.7; 2.6)	0.95	0.56 (−1.5; 2.6)	0.58
Model 2^c^	0.87 (−1.1; 2.9)	0.40	−6.0 (−12; −0.37)	0.037	−0.15 (−2.9; 2.6)	0.91	0.56 (−1.5; 2.6)	0.59
Model 3^d^	0.72 (−1.4; 2.8)	0.50	−5.9 (−12; −0.27)	0.040	−0.27 (−3.0; 2.5)	0.85	0.51 (−1.6; 2.6)	0.63
*P* interaction^e^		0.86		0.48		0.046		
*P* interaction^f^		0.14		0.15		0.51		
*Memory scores*								
Model 1^b^	1.4 (−0.50; 3.4)	0.14	−4.8 (−12; 2.0)	0.16	1.9 (−0.66; 4.4)	0.15	−0.13 (−2.0; 1.8)	0.89
Model 2^c^	1.0 (−0.90; 3.0)	0.23	−4.7 (−12; 2.2)	0.18	1.7 (−0.84; 4.3)	0.19	−0.34 (−2.3; 1.6)	0.73
Model 3^d^	1.2 (−0.77; 3.3)	0.23	−4.8 (−12; 2.1)	0.18	1.9 (0.72; 4.5)	0.15	−0.28 (−2.2; 1.7)	0.78
*P* interaction^e^		0.72		0.55		0.17		
*P* interaction^f^		0.077		0.009		0.76		
*Perceptive-performance scores*								
Model 1^b^	0.077 (−1.7; 1.8)	0.93	−2.1 (−7.7; 3.6)	0.48	1.5 (−0.78; 3.9)	0.19	0.33 (−1.5; 2.2)	0.73
Model 2^c^	−0.36 (−2.2; 1.5)	0.70	−1.9 (−7.6; 3.9)	0.52	1.9 (−0.71; 4.6)	0.15	0.059 (−1.9; 2.0)	0.95
Model 3^d^	−0.25 (−2.1; 1.6)	0.80	−1.9 (−7.6; 3.8)	0.51	2.0 (−0.66; 4.7)	0.14	0.097 (−1.8; 2.0)	0.92
*P* interaction^e^		0.29		0.30		0.54		
*P* interaction^f^		0.021		0.035		0.34		
*Motor scores*								
Model 1^b^	0.81 (−1.1; 2.7)	0.40	−4.2 (−10; 2.0)	0.18	0.54 (−1.6; 2.7)	0.63	−0.71 (−2.8; 1.4)	0.50
Model 2^c^	0.64 (−1.4; 2.7)	0.53	−4.1 (−10; 2.1)	0.19	0.69 (−1.9; 3.3)	0.60	−0.81 (−3.0; 1.3)	0.46
Model 3^d^	0.88 (−1.2; 2.9)	0.40	−4.2 (−10; 1.9)	0.18	0.87 (−1.7; 3.5)	0.51	−0.73 (−2.9; 1.4)	0.51
*P* interaction^e^		0.23		0.60		0.63		
*P* interaction^f^		0.011		0.006		0.098		
*Executive functions, frontal cortex predominance*								
Model 1^b^	1.9 (−0.067; 3.8)	0.059	−7.2 (−13; −1.6)	0.011	1.5 (−0.98; 4.0)	0.24	−0.59 (−2.6; 1.4)	0.57
Model 2^c^	1.5 (−0.43; 3.5)	0.13	−7.1 (−13; −1.5)	0.013	1.4 (−1.3; 4.0)	0.31	−0.76 (−2.8; 1.3)	0.47
Model 3^d^	1.5 (−0.49; 3.6)	0.14	−7.1 (−13; −1.5)	0.014	1.4 (−1.3; 4.0)	0.32	−0.76 (−2.8; 1.3)	0.47
*P* interaction^e^		0.90		0.65		0.22		
*P* interaction^f^		0.025		0.008		0.75		
*Cognitive functions, posterior cortex predominance*								
Model 1^b^	0.46 (−1.5; 2.4)	0.64	−4.2 (−10; 1.6)	0.16	1.9 (0.50; 4.2)	0.12	0.28 (−1.6; 2.2)	0.77
Model 2^c^	−0.063 (−2.0; 1.9)	0.95	−4.0 (−10; 1.9)	0.19	2.3 (−0.22; 4.8)	0.074	−0.047 (−1.9; 1.9)	0.96
Model 3^d^	0.21 (−1.8; 2.2)	0.83	−4.2 (−10; 1.8)	0.17	2.5 (−0.055; 5.1)	0.055	0.048 (−1.9; 2.0)	0.96
*P* interaction^e^		0.29		0.29		0.11		
*P* interaction^f^		0.043		0.013		0.94		

^a^Number of individuals < and ≥0.8 µg cadmium/L: 469 and 107

^b^Model 1: Separate regression analysis for each element, adjusted for examiner, child sex, age at testing (years), and maternal age (<25 years, ≥25 to <35 years, and >35 years), parity (primiparous/multiparous), marital status (married-engaged/single), education (≤6 years, >6 to ≤12 years, >12 years), and tobacco smoking (never/ever)

^c^Model 2: Combined analysis of all elements, adjusted as Model 1

^d^Model 3: Model 2, additionally adjusted for urinary lead

^e^Addition of a multiplicative interaction in Model 3 between log_2_ urinary cadmium or log_2_ urinary cadmium or log_2_ urinary selenium with child sex (boy = 0/girl = 1)]

^f^Addition of a multiplicative interaction in Model 3 between log_2_ urinary cadmium or log_2_ urinary cadmium or log_2_ urinary selenium with tobacco smoking (never = 0/ever = 1)]

In sensitivity analyses, we additionally adjusted Model 3 for covariates which may have been in the causal pathway between the children’s prenatal cadmium exposure or nutritional status and their general cognitive function at 4 years of age (Supplemental Table S1). Additional adjustment for either birth weight, gestational age at birth (<37 and ≥37 weeks of gestation), or weight-for-age at 4 years of age had essentially no impact on the inverse association between urinary cadmium ≥0.8 µg/L and the children’s general cognitive score (Supplemental Table S1). However, restricting the analyses to children born at term (≥37 weeks of gestation) increased the estimate of urinary cadmium ≥0.8 µg/L by about 30 % (*p* = 0.007). Additional adjustment for duration of breastfeeding (missing data for 5 % of the children; n = 549) decreased the estimate of urinary cadmium ≥0.8 µg/L by about 7 % and the association was no longer significant (*p* = 0.059). The positive association between urinary selenium and the general cognitive score increased by 12 % after additional adjustment for duration of breast feeding (Supplemental Table S1).

We examined if there was any interactions between the mothers’ urinary cadmium or urinary selenium and child sex in relation to the general cognitive score. The multiplicative interaction term was neither significant for the association <0.8 µg Cd/L, nor the association ≥0.8 µg Cd/L (Table 2: Model 3; *p* > 0.10). For urinary selenium, the interaction was close to significant (*p* = 0.13), indicating a more pronounced effect in girls. Finally, we assessed if maternal smoking (never/ever) modified our studied association of the mother’s urinary cadmium and selenium with the children’s general cognitive score. Smoking showed an interaction with both the positive association between urinary cadmium concentrations <0.8 µg/L and the general cognitive score (*p* = 0.036) and with the inverse association between urinary cadmium concentrations ≥0.8 µg/L and the general cognitive score (*p* = 0.014; Table 2). In general, the same results were obtained in relation to all the different sub-scales of the children’s general cognitive score. Stratification by the mothers’ smoking status (Table 3), showed that the associations with cadmium were restricted to children born to mothers classified as smokers.

**Table 3:** Regression analysis of maternal urinary concentrations of cadmium (spline knot at 0.8 µg Cd/L) with their children’s general cognitive score as well as with different sub-scales of the general cognitive score at 4 years of age, stratified by maternal smoking during pregnancy

Outcomes	Never smokers				Ever smokers			
	β1 (95 % CI)^a,c ^	*p*	β2 (95 % CI)^a,c ^	*p*	β1 (95 % CI)^b,c ^	*p*	β2 (95 % CI)^b,c ^	*p*
*General cognitive scores*								
Urinary Cd (log_2_)	−1.0 (−3.5; 1.4)	0.42	0.16 (−7.1; 7.5)	0.97	3.4 (−0.091; 6.9)	0.056	−12 (−21; −2.9)	0.010
Verbal scores								
Urinary Cd (log_2_)	−0.16 (−2.6; 2.3)	0.90	−1.1 (−8.2; 6.0)	0.76	3.8 (0.15; 7.5)	0.041	−13 (−24; −2.4)	0.017
Memory scores								
Urinary Cd (log_2_)	−0.22 (−2.8; 2.3)	0.87	3.5 (−4.2; 11)	0.37	2.7 (−0.62; 6.0)	0.11	−13 (−24; −2.4)	0.016
*Perceptive-performance scores*								
Urinary Cd (log_2_)	−1.7 (−4.1; 0.66)	0.16	2.8 (−5.2; 11)	0.50	2.3 (−0.89; 5.5)	0.16	−6.6 (−15; 1.3)	0.10
*Motor scores*								
Urinary Cd (log_2_)	−0.57 (−3.1; 2.0)	0.67	2.8 (−5.7; 11)	0.52	2.8 (−0.53; 6.1)	0.099	−11 (−19; −2.9)	0.008
*Executive functions, frontal cortex predominance*								
Urinary Cd (log_2_)	−0.39 (−2.9; 2.1)	0.76	−0.71 (−8.5; 7.1)	0.86	4.7 (1.1; 8.3)	0.010	−13 (−21; −4.7)	0.002
*Cognitive functions, posterior cortex predominance*								
Urinary Cd (log_2_)	−1.4 (−3.9; 1.1)	0.26	2.5 (−5.1; 10)	0.52	2.7 (−0.82; 6.2)	0.13	−11 (−20; −0.95)	0.032

^a^Spline knot at 0.8 µg/L, number of individuals < and ≥0.8 µg cadmium/L: 318 and 60

^b^Spline knot at 0.8 µg/L, number of individuals < and ≥ 0.8 µg cadmium/L: 151 and 46

^c^Adjusted for examiner, child sex, age at testing (years), and maternal age (<25 years, ≥25 to <35 years, and >35 years), parity (primiparous/multiparous), marital status (married-engaged/single), education (≤6 years, >6 to ≤12 years, >12 years), and urinary concentrations of selenium, iodine, and lead

## Discussion

The present findings support a few previous studies indicating that elevated cadmium exposure during pregnancy may be inversely associated with children’s cognitive function measured at pre-school age. We found a significant interaction between the mother’s urinary cadmium concentration and smoking status, and further stratification showed that only children born to mothers who smoked during pregnancy, and also had higher exposure levels, appeared affected. We also observed a marginal beneficial effect of the mothers’ selenium status during pregnancy on the children’s cognitive function, but it did not markedly affect the inverse association of cadmium exposure with cognitive function.

To our knowledge, only three previous studies have observed an association between women’s cadmium exposure during pregnancy and cognitive function of their children [4, 5, 7]. A small study including 4.5 year-old children (n = 106) residing close to a metal smelter in central China [4] suggested that children with cord blood cadmium concentrations above the median of 0.60 µg/L had significantly lower full scale and performance IQ, but not verbal IQ, compared to those with cadmium concentrations below the median. In a small South Korean study (n = 119), maternal blood cadmium concentrations during pregnancy were inversely associated with performance IQ, but not with verbal or full-scale IQ, at 5 years of age [7]. In a much larger prospective study in rural Bangladesh (n = 1305), maternal urinary cadmium concentrations in early pregnancy were inversely associated with the children’s full scale, performance and verbal IQ at 5 years of age [5]. Importantly, the above mentioned cadmium-related effects were independent of tobacco smoking as none of the mothers smoked [4, 5, 7]. In the present study, we found no significant associations in never-smokers, who, however, had much lower cadmium exposure (median urinary cadmium 0.40 µg/L and blood cadmium 0.29 µg/L) [29] than in the mother’s in China (median blood cadmium 1.8 µg/L), South Korea (mean blood cadmium 1.5 µg/L) and Bangladesh (median urinary cadmium 0.63 µg/L) ([4, 5, 7]. The higher cadmium exposure in the three latter cohorts were most likely due to their rice-based diet (in the Chinese study also pollution from the industrial activities), as rice is known to take up much cadmium from the soil [30]. The diet in the present study was more typical of a Western diet [31]. Obviously, the role of smoking in cadmium toxicity is complex, as it is a major source of cadmium [16], but also of many other toxic substances. Thus, we cannot totally exclude the fact that cadmium may be a proxy of a combined toxicity of multiple toxicants in tobacco smoke on child development.

We hypothesize that the positive associations between lower maternal urinary cadmium concentrations (<0.8 µg/L) and the different developmental outcome scores, although not statistically significant, is due to residual confounding as the associations gradually decreased after we adjusted for multiple potential confounders. Besides cadmium exposure from tobacco smoking, exposure also occurs via food. The cadmium concentrations in food vary considerably, but fiber-rich foods of plant origin usually contribute the most to the human intake [32, 33]. High cadmium concentrations are also found in various seafood and offal. Thus, cadmium may in the present study serve as an indicator of a healthy food pattern with a high intake of whole grain cereals, roots, vegetables, and shellfish, leading to intake of multiple nutrients and antioxidants, all of which are beneficial for neurodevelopment [34, 35]. Indeed, adjusting for the women’s selenium and iodine status markedly decreased these positive associations by about 20–45 % and even changed some of them from positive to negative. However, at higher exposure levels, i.e. at maternal urinary cadmium concentrations ≥0.8 µg/L, the negative impact of cadmium on cognitive function appeared to exceed the beneficial effects of the healthy food pattern. In support for this interpretation, the adjustments for selenium and iodine were less influential (all <12 %) at the higher cadmium exposure levels. This clearly highlights the importance of considering various nutritional factors, including essential nutrients, when evaluating adverse health effects of environmental pollutants present in food.

The exact mechanisms of cadmium-induced neurotoxicity are still unresolved. Despite the fact that women’s cadmium concentrations in blood during pregnancy accumulate in the placenta, it is possible that the small fraction which passes over to the fetus may have a direct toxic effect on neurodevelopment. In experimental studies, cadmium-induced neuronal toxicity has been proposed to be mediated via (i) induction of reactive oxygen species [36, 37]; (ii) impaired differentiation [38] and (iii) neurochemical changes (i.e. disruption of calcium signaling or changes in neurotransmitters) [39–41]. The experimental studies showing a potential impact of cadmium on neurotransmitters are supported by findings in a cross-sectional study of European children [42], in which the children’s cadmium exposure affected their dopamine metabolism. Whether this is true also for prenatal exposure is not known. Moreover, we have previously reported a positive association between cadmium exposure and a marker of oxidative DNA damage in both pregnant women and infants [43, 44]. Another alternative hypothesis is that cadmium can indirectly affect neurodevelopment via its accumulation in placenta, which may cause impaired transfer of the essential nutrient zinc [45] or of thyroid hormones [46], or alter the fetal programming by affecting the DNA methylation during gestation [47].

The present finding that the women’s selenium status during pregnancy appeared to be beneficial for early cognitive development is supported by a recent prospective study in rural Bangladesh, including 750 mothers and their children at 1.5 years of age [14]. In that study, the mother’s blood selenium concentrations were positively associated with the children’s psychomotor development and language comprehension. In the present cohort the associations were slightly weaker, probably because most of the women appeared to have an adequate selenium status (mean whole blood selenium 112 µg/L; range 73–158 µg/L; n = 50; unpublished data), while 60 % of the Bangladeshi women were selenium deficient [14]. Still, it is noteworthy that the children’s neurodevelopment seemed to improve even within the range of women’s adequate selenium status. Interestingly, the positive association between selenium and children’s cognitive function appeared to be more pronounced in girls than in boys. This was also the case for the effects on psychomotor development in the Bangladeshi children, while the opposite pattern was observed for language comprehension [14]. The observed sex-differences might be related to interactions between selenium status, sex hormone secretion, and thyroid hormones previously observed in young women [48]. Also, experimental studies in mice have shown sex-specific differences in the expression of type I iodothyronine deiodinases [49]. However, the exact mechanisms behind these findings remain to be elucidated.

Selenium may indirectly affect brain development via its involvement in selenoproteins, i.e. iodothyronine deiodinases (DIOs), glutathione peroxidases (GPxs) and thioredoxin reductase, which play a vital role in thyroid hormone biosynthesis and thyroid gland function [50, 51]. During pregnancy, normal thyroid function is important for the fetal neurological development. In experimental studies on rats, selenium deficiency caused an increase in plasma thyroxine (T4) and a decrease in tri-iodothyronine (T3; active form) [52]. Besides selenium’s role in thyroid hormone function, experimental studies have also indicated that selenium can influence the levels of brain-derived neurotrophic factor (BDNF) [53, 54], a potent modulator of neuronal development in the central nervous system [55], and the levels of myelin basic protein, a marker of myelination [53].

Despite the documented importance of adequate iodine status for early-life neurodevelopment [56], we observed no association between the mothers’ urinary iodine concentrations and the children’s cognitive function, not even when comparing children born to mothers classified as having an inadequate iodine status (urinary concentrations <150 µg/L [18]; 36 % of the women) to those being born to mothers with an adequate iodine status (≥150 µg/L; data not shown). Our null findings are supported by a recent Dutch study [12]. Probably, the identified iodine deficiency was not enough frequent and severe to affect neurodevelopment. There is a large intra-individual day-to-day variation in urinary iodine [57], and, therefore, repeated sampling would likely give more reliable information than a single spot urine sample, especially for identification of long-term deficiency.

The mother’s urinary lead concentrations were not associated with cognitive function and had only marginal impact on our studied associations. Similarly, in a recent Spanish study where the mothers had slightly higher urinary lead concentrations during the first and third trimester (median 3.4 and 3.6 µg/L, respectively) no associations were observed with the children’s cognitive function at 4 years of age [7]. This is puzzling as the European Food and Safety authority has, based on several studies showing that lead exposure (blood lead) impairs neurodevelopment, concluded that there is no threshold for lead-induced neurotoxicity [58]. Possibly, urinary lead is not as optimal a biomarker as blood lead. Urinary lead has been suggested as an alternative to blood lead due to its relation to concentrations in plasma [59], but the half-life of lead in plasma is much shorter than that in whole blood, making it more difficult to use a single spot-urine sample as a marker of long-term exposure.

The main strengths of this study include the prospective population-based design, wide range of women’s cadmium exposure and selenium and iodine status during pregnancy, and available information regarding multiple potential confounders. The women’s cadmium exposure was assessed by concentrations in urine, using a highly sensitive and reliable ICPMS method. Unlike urinary lead, urinary cadmium is commonly used as a biomarker of chronic cadmium exposure as it is influenced by the body burden of cadmium and proportional to the concentration in the kidney; where cadmium accumulates with a half-life of 10–30 years [16]. Moreover, maternal urinary cadmium concentrations have previously been shown to be associated with the cadmium concentrations in cord blood and placenta as well as with the cadmium concentrations in infant urine [45, 60], suggesting that this biomarker to some extent also captures the children’s early-life exposure to cadmium. A limitation of our study is the incomplete information regarding some covariates, resulting in exclusion of 53 children (about 8 %) from the statistical analyses, despite having information on both the different exposures and outcomes. However, we did not find any significant differences in either of the exposures or outcomes between the children that were included and those that were excluded due to incomplete information on covariates. Secondly, we lack information concerning some potential important confounders such as measures of the caring environment in which the child was raised [e.g. Home Observation for Measurement of the Environment (HOME)], nutritional status of the children (i.e. serum ferritin), and potential illnesses of the children. Finally, besides exploring associations between the different biomarkers and the children’s general cognitive score, we also assessed potential associations with the seven different sub-scales of the general cognitive score, raising concern about multiple testing. An application of Bonferroni correction to take into account multiple comparisons is inappropriate in this case given that we are studying outcomes (sub-scales) which are highly correlated [61]. However, our main results refer to general cognitive function rather than to sub-scales.

In conclusion, the present findings suggest that elevated cadmium exposure during pregnancy may be inversely associated with children’s cognitive function measured at pre-school age. The role of smoking remains unclear as it is both an important source of cadmium exposure related to neurotoxicity as well as a source of residual confounding. In contrast to early-life cadmium exposure, adequate selenium status during early-life appeared to be beneficial for cognitive development. Further large-scale prospective studies in other populations are needed to confirm the present findings and to understand the biological basis of the observed associations.

## Electronic supplementary material

Below is the link to the electronic supplementary material.

Supplementary material 1 (DOCX 18 kb)
