# Supplementary material for: Impact of prenatal exposure to cadmium on cognitive development at preschool age and the importance of selenium and iodine
Source: Eur J Epidemiol. 2016 May 4;31(11):1123–34. doi: 10.1007/s10654-016-0151-9 (PMC5206289; doi:10.1007/s10654-016-0151-9)
Supplement: Supplementary file 1 — Supplementary material 1 (DOCX 18 kb) [file 10654_2016_151_MOESM1_ESM.docx]

**Supplemental Material**

**Impact of prenatal exposure to cadmium on cognitive development at preschool age and the importance of selenium and iodine**

Maria Kippler^a^, Matteo Bottai^a^, Vaggelis Georgiou^b^, Katerina Koutra^b^, Georgia Chalkiadaki^b^, Mariza Kampouri^b^, Andriani Kyriklaki^b^, Marina Vafeiadi^b^, Eleni Fthenou^b^, Maria Vassilaki^b^, Manolis Kogevinas^c,d,e^, Marie Vahter ^a^, Leda Chatzi^b^

^a^Institute of Environmental Medicine, Karolinska Institutet, Box 210, SE-171 77, Stockholm, Sweden;

^b^Department of Social Medicine, Faculty of Medicine, University of Crete, Heraklion, 71003, Crete, Greece

^c^Centre for Research in Environmental Epidemiology (CREAL), Barcelona, E-08003, Spain

^d^Municipal Institute of Medical Research (IMIM-Hospital del Mar), Barcelona, E-08003, Spain

^e^National School of Public Health, Athens, 11521, Greece

*Correspondence addressed to:

Maria Kippler, Institute of Environmental Medicine, Karolinska Institutet, Box 210, SE-171 77, Stockholm, Sweden. Telephone: +46 8 524 874 07. Telefax: +46 8 33 69 81. E-mail: Maria.Kippler@ki.se

**Table S1.** Regression analysis of maternal urinary concentrations of cadmium (spline knot at 0.8 µg Cd/L), selenium, and iodine (log_2_-tranfomed) with their children’s general cognitive score at 4 years of age.

|  |  | **Urinary Cd (log_2_; spline knot at 0.8 µg/L)** | | | | **Urinary Se (log_2_)** | | **Urinary iodine (log_2_)** | |
| --- | --- | --- | --- | --- | --- | --- | --- | --- | --- |
| **Outcomes** | n | β1 (95% CI) | *p* | β2 (95% CI) | *p* | β (95% CI) | *p* | β (95% CI) | *p* |
| **General cognitive scores** | | |  |  |  |  |  |  |  |
| Model A^1,2^ | 574 | 0.85(-1.1; 2.8) | 0.40 | -6.0 (-12; -0.32) | 0.038 | 2.2 (-0.38; 4.8) | 0.094 | -0.17 (-2.1; 1.8) | 0.86 |
| Model B^1,3^ | 568 | 0.83 (-1.2; 2.8) | 0.41 | -6.2 (-12; -0.45) | 0.035 | 2.4 (-0.26; 5.0) | 0.078 | -0.053 (-2.0; 1.9) | 0.96 |
| Model C^1,4^ | 494 | 1.6 (-0.54; 3.6) | 0.15 | -8.6 (-15; -2.3) | 0.007 | 2.1 (-0.64; 4.9) | 0.13 | -0.51 (-2.6; 1.6) | 0.63 |
| Model D^1,5^ | 549 | 0.72 (-1.3; 2.7) | 0.48 | -5.7 (-12; 0.23) | 0.059 | 2.5 (-0.20; 5.1) | 0.070 | -0.080 (-2.1; 1.9) | 0.94 |
| Model E^1,6^ | 574 | 0.75 (-1.2; 2.7) | 0.46 | -6.3 (-12; -0.48) | 0.034 | 2.3 (-0.32; 4.9) | 0.086 | -0.15 (-2.1; 1.8) | 0.88 |

^1^Combined analysis of all elements, adjusted for examiner, child sex, age at testing (years), and maternal age (<25 y, ≥25 to <35 years, and >35 years), parity (primiparous/multiparous), marital status (married-engaged/single), education (≤6 years, >6 years to ≤12 years, >12 years), tobacco smoking (never/ever), urinary lead (log_2_-tranformed)

^2^Additionally adjusted for birth weight.

^3^Additionally adjusted for gestational age at birth (<37 weeks and ≥37 weeks of gestation).

^4^Restricted to children born at term (≥37 weeks of gestation).

^5^Additionally adjusted for duration of breast feeding (no, 1-6 months or >6 months).

^6^Additionally adjusted for weight-for-age z-scores at 4 years of age.
